# The Role of MicroRNA in the Myocarditis: a Small Actor for a Great Role

**DOI:** 10.1007/s11886-023-01888-5

**Published:** 2023-06-03

**Authors:** Cristina Chimenti, Michele Magnocavallo, Giampaolo Vetta, Maria Alfarano, Giulia Manguso, Francesco Ajmone, Federico Ballatore, Jacopo Costantino, Piera Ciaramella, Paolo Severino, Fabio Miraldi, Carlo Lavalle, Carmine Dario Vizza

**Affiliations:** 1 Clinical, Anestesiologic and Cardiovascular Sciences, La Sapienza University of Rome, Rome, Italy; 2 Cardiology Division, Arrhythmology Unit, S. Giovanni Calibita Hospital, Isola Tiberina, Rome, Italy; 3 Department of Clinical and Experimental Medicine, Cardiology Unit, University of Messina, Mesina, Italy

**Keywords:** MicroRNA, Myocarditis, Experimental myocarditis, Pathogenesis, Diagnosis

## Abstract

**Purpose of Review:**

Myocarditis is an inflammation of the myocardium secondary to a variety of agents such as infectious pathogens, toxins, drugs, and autoimmune disorders. In our review, we provide an overview of miRNA biogenesis and their role in the etiology and pathogenesis of myocarditis, evaluating future directions for myocarditis management.

**Recent Findings:**

Advances in genetic manipulation techniques allowed to demonstrate the important role of RNA fragments, especially microRNAs (miRNAs), in cardiovascular pathogenesis**.** miRNAs are small non-coding RNA molecules that regulate the post-transcriptional gene expression. Advances in molecular techniques allowed to identify miRNA’s role in pathogenesis of myocarditis.

**Summary:**

miRNAs are related to viral infection, inflammation, fibrosis, and apoptosis of cardiomyocytes, making them not only promising diagnostic markers but also prognostics and therapeutic targets in myocarditis. Of course, further real-world studies will be needed to assess the diagnostic accuracy and applicability of miRNA in the myocarditis diagnosis.

## Introduction

Myocarditis is an inflammation of the myocardium secondary to a variety of agents such as infectious pathogens, toxins, drugs and autoimmune disorders [1]. Myocarditis can self-resolve, cause sudden cardiac death, or lead to dilated cardiomyopathy [2–6]. The real disease burden is unknown due to the challenge of achieving a definite diagnosis in many patients [3]. Myocarditis can often initially simulate an a acute myocardial infarction with non-obstructive coronary arteries (MINOCA), which occurs in approximately 10–20% of patients with an initial diagnosis of myocardial infarction [1]. Frequently, the diagnosis of myocarditis is performed using the Lake Louise criteria at cardiac magnetic resonance (CMR), which is not yet widely available [7]. Endomyocardial biopsy is the gold standard for diagnosis, but it is not always available and employed [2]. Therefore, broadly available and accurate diagnostic means for the prompt detection of acute myocarditis are not currently available. Advances in genetic manipulation techniques allowed to demonstrate the important role of RNA fragments, especially microRNAs, in cardiovascular pathogenesis [8]. MicroRNAs (miRNAs) are small non-coding RNA molecules that regulate the post-transcriptional expression of genes [9]. They are epigenetic regulators of cardiac function, participate in almost all aspects of cardiac physiology and pathology, and are involved in both the etiology and pathogenesis of myocarditis [10]. Advances in molecular techniques allowed to identify myocarditis miRNAs and their mechanisms of action [11]. miRNAs in myocarditis are related to viral infection, inflammation, fibrosis, and apoptosis of myocardiocytes, making them promising diagnostic markers and prognostic and therapeutic targets in myocarditis [12]. In our review, we provide an overview of miRNA biogenesis and their role in the etiology and pathogenesis of myocarditis. Finally, some current perspectives and future directions for myocarditis management are suggested.

## MicroRNA Biogenesis and Function

In recent years, the role of miRNAs, mainly in post-transcriptional regulation, was increasingly studied [12]. miRNAs are non-coding RNAs of 16–26 nucleotides and can bind to the 3′-untranslated region (UTR) of target mRNAs to control their translation [12]. Over 2600 miRNAs were studied recently in human beings, although their function was not fully understood [13]. More than 60% of human protein-coding genes are regulated by miRNAs [13]. miRNAs play a key role in all tissues and in all stages of development by regulating cell differentiation, proliferation, and survival [13]. The miRNA has an important role not only in post-transcriptional regulation but also in the epigenetic modulation of gene expression under the control of the argonaute 2 (AGO2) protein [14].

**Fig. 1:**
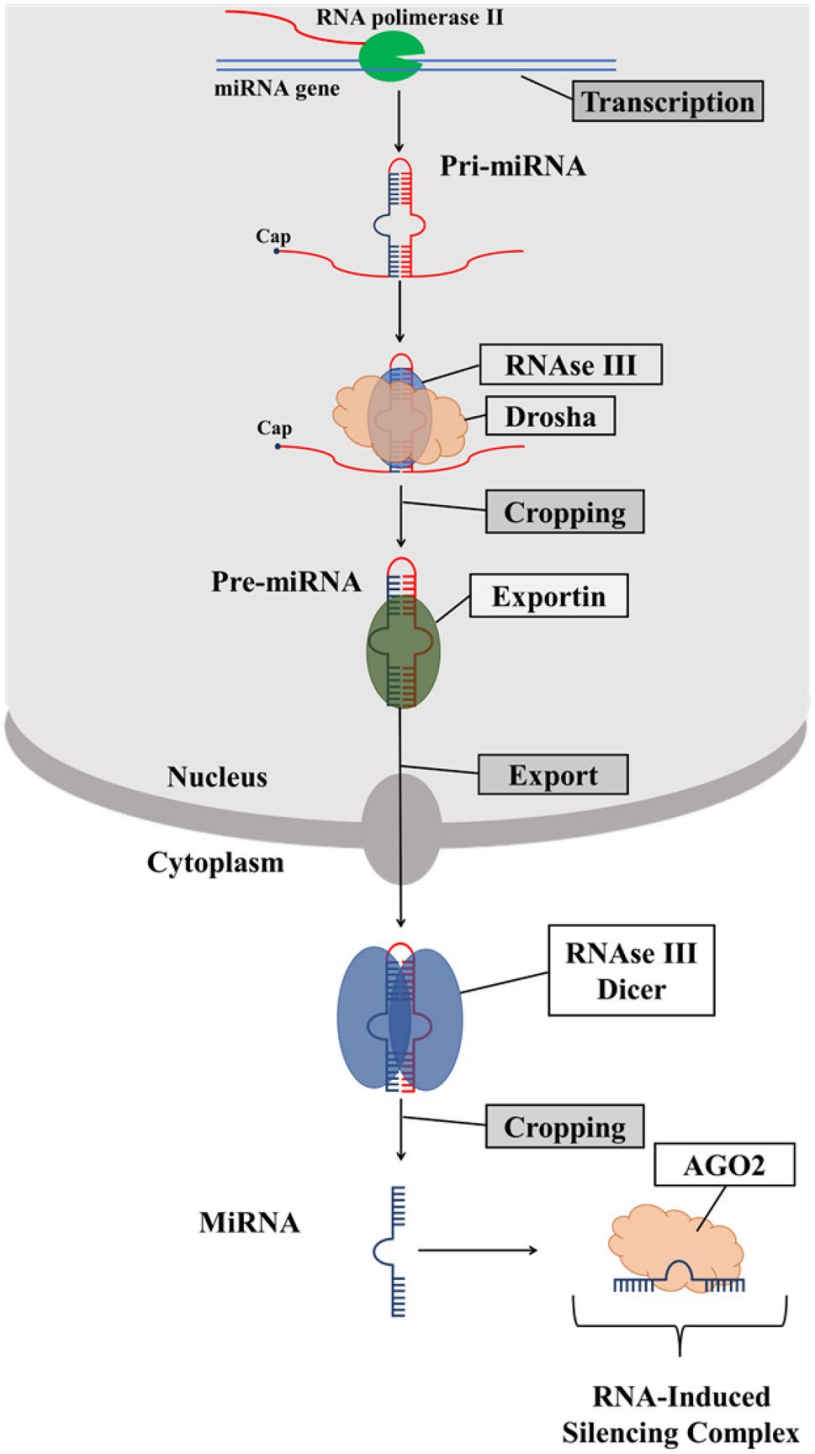
MiRNA biogenesis pathway. AGO2, argonaute 2; miRNA, microRNA; pri-miRNA, primary microRNA

miRNAs are found in intergenic regions, introns, and polycistronic sites and can be transcribed in clusters or alone (Fig. 1) [15]. Polymerase II transcribes primary miRNAs (pri-miRNAs) with a stem-loop structure (5′ cap and a 3′ poly A tail) like mRNAs [15]. Pri-miRNAs are cleaved by RNase III Drosha into smaller miRNA precursors of 60–70 nucleotides (pre-miRNAs), transported into the cytoplasm by exportin [16]. Here, RNase III dicer further cleaves the pre-miRNAs into short miRNA, which binds to the AGO2 protein, creating RNA-induced silencing complex (RISC) [17]. RISC binding with its 5′ end to the 3′ UTR, 5′ UTR or even to the coding region leads to post-transcriptional gene silencing by inhibition of translational expression or the mRNA degradation [18]. One miRNA influences several miRNAs, and a single mRNA can also be controlled by several miRNAs [18].

## MicroRNAs and Myocarditis

**Fig. 2:**
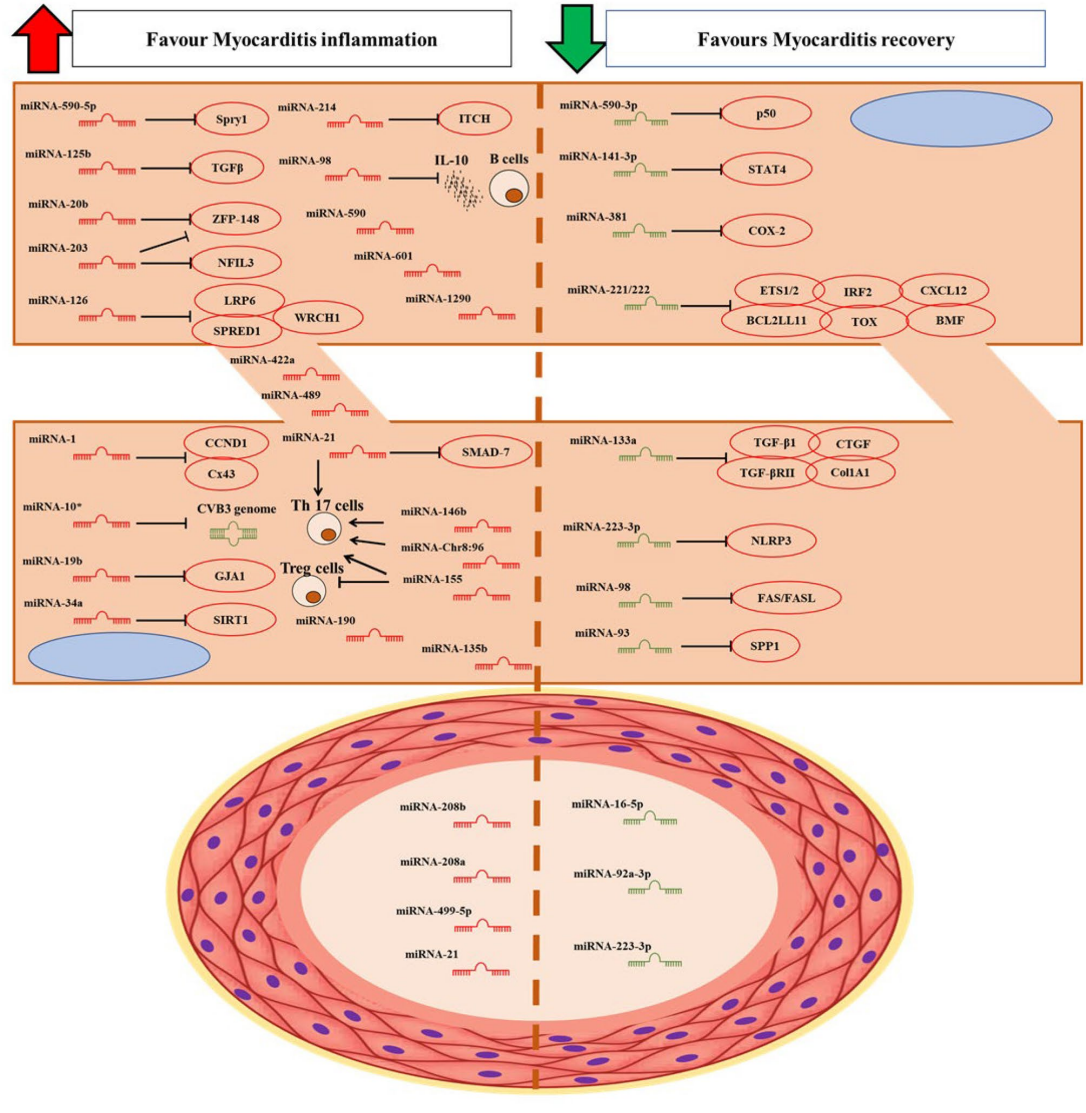
miRNAs involved in myocarditis and their targets. Col1A1, collagen 1A1; CVB3, Coxsackievirus B3; Cx43, connexin 43; miRNA, microRNA; NFIL3, nuclear factor interleukin-3; NLRP3, pyrin domain-containing-3; SPRED1, sprouty-related EVH1 domain containing 1; Spry1, sprouty-1; TGF-β1, transforming growth factor-β1; TGF-βRII, transforming growth factor-β receptor type II; ZFP-148, zinc finger protein-148

In our review, we classified as intracellular the miRNAs detected in cardiac biopsies in patients with myocarditis, and extracellular the miRNAs detected in blood samples. Figure 2 resumes the miRNAs involved in myocarditis and their targets.

## Intracellular MicroRNA

### Myocardium MicroRNAs

miRNA-1 is the most frequent miRNA within the cardiac cell, accounting for 40% of all miRNAs and is transcribed along with miRNA-133, co-regulating myocardial cell proliferation and differentiation [19]. Indeed, miRNA-1 and miRNA-133 favor mesoderm formation from embryonic cells but have opposite roles during subsequent differentiation into cardiac muscle progenitors [19]. Furthermore, miRNA-1 and miRNA-133 play a key role in the repression of non-muscle genes during embryonic cell differentiation. miRNA-1 and 133 also play an important role in cardiomyopathies such as myocarditis [20–23]. Indeed, miRNA-1 has been shown to inhibit post-transcriptional expression of connexin 43 (Cx43), the main cardiac gap junction that modulates cardiac conduction, promoting the myocardiocyte apoptosis and triggering arrhythmias [24]. miRNA-1, miRNA-133a, and miRNA-133b also play an important role in chronic Chagas disease cardiomyopathy by regulating cyclin D1, a cell cycle regulator of cardiomyocyte proliferation [20]. In patients with inflammatory cardiomyopathy, endomyocardial miRNA-133a levels correlate with myocardial inflammation and improved left ventricular function [21]. Indeed, miRNA-133a was associated with reduced fibrosis and myocyte necrosis on endomyocardial biopsy and left ventricle functional recovery, malignant arrhythmias, and heart failure admissions during a mean follow-up of 3 years [21–23]. These effects of miRNA-133a are caused by repression of transforming growth factor-β 1 (TGF-β1), TGF-β receptor type II (TGF-βRII), connective tissue growth factor (CTGF), or collagen 1A1 (Col1A1) [25]. Myosin is the key regulator of muscle strength and contractility and it is expressed by Myh6, Myh7, and Myh7b, which encode related miRNAs that regulate myosin transcription and performance [26]. In adult myocardiocytes, the Myh6 gene co-expresses miRNA-208a, regulating expression of the other two myosins and their miRNAs (Myh7/miR-208b and Myh7b/miR-499) [27]. In viral myocarditis, the level of miRNA-208a was consistently increased in the acute phase compared to the subacute and resolution/chronic phases [27]. In addition, heart-associated miRNA-208b levels during the subacute phase correlated with recovery of systolic left ventricular function in the resolution/chronic phase [27].

### Viral Infection-Related MicroRNAs

miRNAs associated with myocarditis include miRNAs directly involved in viral myocarditis that regulate viral replication or virulence by acting directly on the virus genome or by regulating host responses to viral infection [28]. The star strand of miRNA-10a (miRNA-10a*) could significantly up-regulate the biosynthesis of group B type 3 coxsackievirus (CVB3) through binding to the nt6818-nt6941 sequence of the viral DNA promoting the onset of viral myocarditis [29]. Furthermore, miRNA-10a* was detectable in the Balb/c suckling murine model myocardium, highlighting the influence of miRNA-10a* on CVB3 replication in the myocardiocytes [29]. In addition, miRNA-20b influences the action of zinc finger protein-148 (ZFP-148), a transcription factor that plays an essential role in regulating virus replication, by binding directly to the 3′-UTR and inhibiting its expression [30]. Instead, CVB3 activates ERK1/2 through phosphorylation of the transcription factors ETS-1 and ETS-2, resulting in overexpression of miRNA-126 [31]. Upregulation of miRNA-126 reduces the sprouty-related EVH1 domain containing 1 (SPRED1) by activating ERK1/2 resulting in a positive feedback loop [31]. In addition, miRNA-126 induces GSK-3β, which promotes virus-induced cell death, leading to viral release and virus spread [31]. Instead, miRNA-590-5p is the most highly expressed miRNA in viral vesicles, which promotes viral replication by inhibiting apoptosis through suppression of the antiviral sprouty-1 (Spry1) [32]. In fact, myocardiocytes with increased miRNA-590-5p levels were at a higher risk of infection [32]. The expression of miRNA-223-3p was reduced in the mouse model of experimental autoimmune myocarditis (EAM) compared to the wild type [33]. miRNA-223-3p inhibited pyrin domain-containing-3 (NLRP3) inflammasome formation and promoted regulatory T-cell activation by inhibiting dendritic cell (DC) activation [33]. Administration of DCs with increased expression of miRNA-223-3p was protective against EAM [33]. miRNA-21, miRNA-146b, and miRNA-155 are increased in acute viral myocarditis (VMC) and in the mouse model with CVB3 or *T. cruzi* myocarditis [34•, 35]. Indeed, miRNA-21 and -146b antagonists inhibit Th17 and RORγt expression and reduce myocardial inflammation [36]. Similarly, miRNA-155 antagonists alter Th17/Treg balance by reducing Th17 activation, thereby reducing myocardial inflammatory damage [37]. miRNA-98 promotes the onset of myocarditis by inhibiting IL-10 release, but at the same time, miRNA-98 acts on FAS/FASL by inhibiting myocardial apoptosis [38, 39].

The miRNA-221/-222-targeted ETS1/2, IRF2, BCL2L11, TOX, BMF, and CXCL12 resulted in increased viral replication and inflammation in the mouse model of VMC [40].

Inhibitors of miRNA-221/-222 enhanced cardiac viral load and increased inflammatory cardiac damage; conversely, the increase in miRNA-221/-222 inhibited viral replication and protected against myocardial damage [40]. miRNA-203 promotes viral replication by inhibiting ZFP-148 and favors myocardial apoptosis by acting on the nuclear factor interleukin-3 (NFIL3) [41]. In addition, miRNAs play an important role in myocardial fibrosis in chronic VMC. Indeed, miRNA-21 and miRNA-125b induce myocardial fibrosis by suppressing SMAD-7, a negative regulator of TGF-β signalling [42•].

### MicroRNAs and T cells

miRNAs play an important role in T-cell development and function. Indeed, miRNA deficiency in the early phase of T-cell formation reduces thymocyte survival, and, in the subsequent phases of development, leads to a reduction in the number of peripheral CD4 + T-cells and blocks the development of CD8 + T-cells [43, 44]. Decreased miRNA expression in CD4 + T cells results in a phenotypic switch to Th1 instead of Th2, leading to a reduction in T-reg cells [45, 46]. Indeed, miRNA-21 drives Th1 cell differentiation through modulation of IL-12 release, preserving the Th1/Th2 balance [47]. Downregulation of miRNA-21 induces Th1 cell differentiation, while upregulation of miRNA-21 leads to Th2 cell differentiation [48]. Furthermore, miRNA-21 enhances Th17 differentiation through SMAD-7, a TGF β pathway inhibitor [48]. Conversely, miRNA-155 favors the maintenance of Treg cells and enhances Th1- and Th17-cell-mediated inflammation [49]. Lack of miRNA-155 impairs humoral and T-cell-dependent cellular immunity as well as induces Th2 polarization [50–53]. Blanco-Domínguez et al. found a new miRNA, mmu-miR-721, as a marker of myocarditis in the mouse model, and its human homolog, has-miR-Chr8:96 [54]. Mmu-miR-721 is an upregulated miRNA in mouse models with myocarditis and is produced by Th17 lymphocytes, affecting myocardial inflammation. The diagnostic ability of the detection of hsa-miR-Chr8:96 in plasma to discriminate myocarditis from other conditions was evaluated in different cohorts of patients with different comparators, including myocardial infarction, MINOCA, and autoimmune diseases, and compared to healthy people [54]. The miRNA had a very good diagnostic efficacy in discriminating patients with acute myocarditis from those with myocardial infarction (area under the curve [AUC]: 0.93; 95% CI: 0.88–0.98), from healthy patients (AUC: 0.99; 95% CI: 0.97–1.00), and from MINOCA (AUC: 0.83; 95% CI: 0.72–0.94) together with patients with other Th17-related diseases (rheumatoid arthritis, spondyloarthritis, psoriasis, and multiple sclerosis) (AUC: 0.99; 95% CI: 0.98–1.00) [54]. miRNA maintained its diagnostic value in models after adjustment for age, gender, ejection fraction, and serum troponin level.

## Extracellular Signaling

miRNAs do not only play an intracellular but also an intercellular signalling role via extracellular vesicles such as exosomes, microvesicles, and apoptotic bodies, or as part of protein/lipoprotein complexes [55••, 56]. miRNAs can be released into circulation by active secretion or passive leak from cell membranes, thus constituting the fraction of circulating miRNAs [57, 58]. These miRNAs are remarkably resistant to degradation by RNase and directly influence various physiological and pathological processes [59, 60]. Circulating miRNAs in myocarditis correlate with disease severity and predict prognosis. So far, no single miRNA has been identified as a specific marker of chronic myocarditis. Corsten et al. found increased levels of miRNA-208b and miRNA-499-5p in the plasma of 14 patients with acute CMV, which correlated directly with troponin T levels [27]. Goldberg et al., in a pediatric population, showed that miRNA-21 and miRNA-208a were increased in the acute phase of VMC. Furthermore, miRNA-208a levels in the subacute phase correlated with the degree of recovery of left ventricular systolic function in the resolution phase [28].

## Future Perspectives

**Fig. 3:**
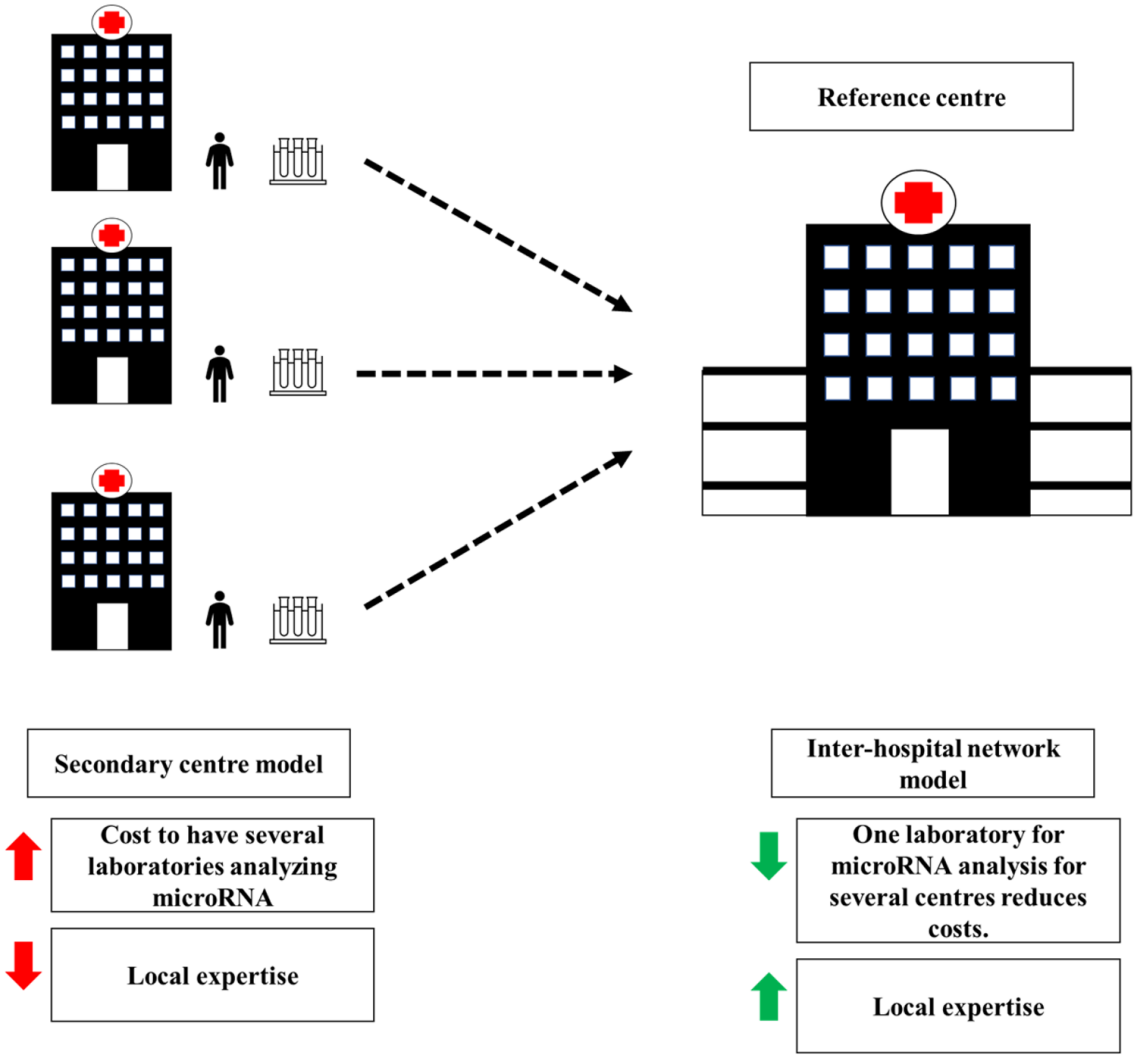
Single-center model and inter-hospital network model with reference center for microRNA analysis

Myocarditis is a complex disease in terms of etiology, diagnosis, and pathogenesis. Recent studies have shown that miRNAs play a key role in the pathogenesis of myocarditis and will become increasingly important in the diagnostic and prognostic management of myocarditis. Myocarditis is often diagnosed according to the Lake Louise criteria on CMR, which is not yet widely available. As the gold standard for diagnosis, endomyocardial biopsy is not always available and employed. Hence, the myocarditis diagnostic criteria should be reassessed in order to make them widely available to centers that do not have access to CMR and endomyocardial biopsy. Although most miRNAs suffer from non-specificity for myocarditis, the mi-RNA has-miR-Chr8:96, discovered by Blanco-Dominìguez et al., and is the first miRNA discovered so far to be specific for myocarditis compared to myocardial infarction, MINOCA, autoimmune inflammatory disease, and healthy subjects [54]. Therefore, has-miR-Chr8:96 might represent a true new diagnostic test for myocarditis that is also accessible to centers without CMR and endomyocardial biopsy that could be included in the diagnostic workup of myocarditis. Of course, further studies will be needed to assess the diagnostic accuracy and applicability of miRNAs in the diagnosis of myocarditis in the real world. Furthermore, there may be numerous obstacles to the widespread use of miRNAs for the diagnosis of myocarditis, such as cost, local expertise, and the impossibility of analysis in all laboratories. The advantage of inter-hospital networks for miRNA analysis could be the solution by allowing secondary centers to take a blood sample to be sent to a reference center for miRNA analysis (Fig. 3). Indeed, miRNA assessment can be performed either on patient’ s plasma or serum and biological samples can be easily collected locally and stored at − 80 °C until the shipping to the referral lab. No specific preparation of samples is necessary, and the results can be available in few days. It can be hypothesized that a blood sample will be collected as soon as the clinical suspicion of myocarditis is done and immediately shipped to the lab, so that the result will be available soon after patient admission to the hospital.

## Conclusions

The recent literature has highlighted the important role that miRNAs play in the pathology of myocarditis and their possible role in diagnosis and prognosis. However, the precise and accurate roles of miRNAs in myocarditis have not yet been fully elucidated. Guidelines on the most appropriate design and analysis methods to increase the replicability of studies on circulating miRNAs are scarce. Likewise, more multicenter studies on large cohorts are needed to confirm the reliability of miRNAs as diagnostic and prognostic markers that would allow for more evidence than the preliminary data we currently have.

## Abbreviations

AGO2: Argonaute 2
AUC: Area under the curve
CMR: Cardiac magnetic resonance
Col1A1: Collagen 1A1
CTGF: Connective tissue growth factor
CVB3: Coxsackievirus B3
Cx43: Connexin 43
DC: Dendritic cell
EAM: Experimental autoimmune myocarditis
MINOCA: Myocardial infarction with non-obstructive coronary arteries
miRNA: MicroRNA
miRNA-10a*: Star strand of miRNA-10a
NFIL3: Nuclear factor interleukin-3
NLRP3: Pyrin domain-containing-3
pri-miRNA: Primary microRNA
RISC: RNA-induced silencing complex
SPRED1: Sprouty-related EVH1 domain containing 1
Spry1: Sprouty-1
TGF-β1: Transforming growth factor-β1
TGF-βRII: Transforming growth factor-β receptor type II
UTR: Untranslated region
VMC: Viral myocarditis
ZFP-148: Zinc finger protein-148

## References

[1] Myocarditis BC (2022). N Engl J Med.

[2] Sagar S, Liu PP, Cooper LT (2012). Myocarditis Lancet.

[3] Caforio ALP, Pankuweit S, Arbustini E, Basso C, Gimeno-Blanes J, Felix SB, Fu M, Heliö T, Heymans S, Jahns R, et al. Current state of knowledge on aetiology, diagnosis, management, and therapy of myocarditis: a position statement of the European Society of Cardiology working group on myocardial and pericardial diseases. Eur Heart J. 2013;34:2636–2648, 2648a–2648d. 10.1093/eurheartj/eht210. 10.1093/eurheartj/eht210 23824828

[4] Chimenti C, Scopelliti F, Vulpis E, Tafani M, Villanova L, Verardo R, De Paulis R, Russo MA, Frustaci A (2015). Increased oxidative stress contributes to cardiomyocyte dysfunction and death in patients with Fabry disease cardiomyopathy. Hum Pathol.

[5] Frustaci A, Sabbioni E, Fortaner S, Farina M, del Torchio R, Tafani M, Morgante E, Ciriolo MR, Russo MA, Chimenti C (2012). Selenium- and zinc-deficient cardiomyopathy in human intestinal malabsorption: preliminary results of selenium/zinc infusion. Eur J Heart Fail.

[6] Chimenti C, Russo A, Pieroni M, Calabrese F, Verardo R, Thiene G, Russo MA, Maseri A, Frustaci A (2004). Intramyocyte detection of Epstein-Barr virus genome by laser capture microdissection in patients with inflammatory cardiomyopathy. CCirculation.

[7] Friedrich MG, Sechtem U, Schulz-Menger J, Holmvang G, Alakija P, Cooper LT, White JA, Abdel-Aty H, Gutberlet M, Prasad S (2009). Cardiovascular magnetic resonance in myocarditis: a JACC White paper. J Am Coll Cardiol.

[8] Heymans S, Eriksson U, Lehtonen J, Cooper LT (2016). The quest for new approaches in myocarditis and inflammatory cardiomyopathy. J Am Coll Cardiol.

[9] Zhou S-S, Jin J-P, Wang J-Q, Zhang Z-G, Freedman JH, Zheng Y, Cai L (2018). MiRNAS in cardiovascular diseases: potential biomarkers, therapeutic targets and challenges. Acta Pharmacol Sin.

[10] Fung G, Luo H, Qiu Y, Yang D, McManus B (2016). Myocarditis. Circ Res.

[11] Pollack A, Kontorovich AR, Fuster V, Dec GW (2015). Viral myocarditis–diagnosis, treatment options, and current controversies. Nat Rev Cardiol.

[12] Bartel DP (2004). MicroRNAs: Genomics, bogenesis, mechanism, and function. Cell.

[13] Johnson JL (2019). Elucidating the contributory role of microRNA to cardiovascular diseases (a review). Vascul Pharmacol.

[14] Li B, Meng X, Zhang L (2019). MicroRNAs and cardiac stem cells in heart development and disease. Drug Discov Today.

[15] Borchert GM, Lanier W, Davidson BL (2006). RNA polymerase III transcribes human microRNAs. Nat Struct Mol Biol.

[16] Ruby JG, Jan CH, Bartel DP (2007). Intronic microRNA precursors that bypass Drosha processing. Nature.

[17] Bartel DP (2009). MicroRNAs: target recognition and regulatory functions. Cell.

[18] Small EM, Olson EN (2011). Pervasive roles of MicroRNAs in cardiovascular biology. Nature.

[19] Rao PK, Toyama Y, Chiang HR, Gupta S, Bauer M, Medvid R, Reinhardt F, Liao R, Krieger M, Jaenisch R (2009). Loss of cardiac MicroRNA-mediated regulation leads to dilated cardiomyopathy and heart failure. Circ Res.

[20] Yan B, Wang H, Tan Y, Fu W. MicroRNAs in cardiovascular disease: small molecules but big roles. CTMC. 2019;19;1918–1947.10.2174/1568026619666190808160241. 10.2174/1568026619666190808160241 31393249

[21] Ferreira LRP, Frade AF, Santos RHB, Teixeira PC, Baron MA, Navarro IC, Benvenuti LA, Fiorelli AI, Bocchi EA, Stolf NA (2014). MicroRNAs MiR-1, MiR-133a, MiR-133b, MiR-208a and MiR-208b are dysregulated in chronic Chagas disease cardiomyopathy. Int J Cardiol.

[22] Besler C, Urban D, Watzka S, Lang D, Rommel K-P, Kandolf R, Klingel K, Thiele H, Linke A, Schuler G (2016). Endomyocardial MiR-133a levels correlate with myocardial inflammation, improved left ventricular function, and clinical outcome in patients with inflammatory cardiomyopathy: endomyocardial MiR-133a levels in inflammatory cardiomyopathy. Eur J Heart Fail.

[23] Xu H-F, Ding Y-J, Shen Y-W, Xue A-M, Xu H-M, Luo C-L, Li B-X, Liu Y-L, Zhao Z-Q (2012). MicroRNA- 1 represses Cx43 expression in viral myocarditis. Mol Cell Biochem.

[24] Castoldi G, di Gioia CRT, Bombardi C, Catalucci D, Corradi B, Gualazzi MG, Leopizzi M, Mancini M, Zerbini G, Condorelli G (2012). MiR-133a regulates collagen 1A1: potential role of MiR-133a in myocardial fibrosis in angiotensin II-dependent hypertension. J Cell Physiol.

[25] Shan H, Zhang Y, Lu Y, Zhang Y, Pan Z, Cai B, Wang N, Li X, Feng T, Hong Y (2009). Downregulation of MiR-133 and MiR-590 contributes to nicotine-induced atrial remodelling in canines. Cardiovasc Res.

[26] Duisters RF, Tijsen AJ, Schroen B, Leenders JJ, Lentink V, van der Made I, Herias V, van Leeuwen RE, Schellings MW, Barenbrug P (2009). MiR-133 and MiR-30 regulate connective tissue growth factor: implications for a role of MicroRNAs in myocardial matrix remodeling. Circ Res.

[27] van Rooij E, Quiat D, Johnson BA, Sutherland LB, Qi X, Richardson JA, Kelm RJ, Olson EN (2009). A family of MicroRNAs encoded by myosin genes governs myosin expression and muscle performance. Dev Cell.

[28] Corsten MF, Dennert R, Jochems S, Kuznetsova T, Devaux Y, Hofstra L, Wagner DR, Staessen JA, Heymans S, Schroen B (2010). Circulating nicroRNA-208b and microRNA-499 reflect myocardial damage in cardiovascular disease. Circ Cardiovasc Genet.

[29] Goldberg L, Tirosh-Wagner T, Vardi A, Abbas H, Pillar N, Shomron N, Nevo-Caspi Y, Paret G (2018). Circulating microRNAs: a potential biomarker for cardiac damage, inflammatory response, and left ventricular function recovery in pediatric viral myocarditis. J of Cardiovasc Trans Res.

[30] Tong L, Lin L, Wu S, Guo Z, Wang T, Qin Y, Wang R, Zhong X, Wu X, Wang Y (2013). MiR-10a* up-regulates Coxsackievirus B3 biosynthesis by targeting the 3D-coding sequence. Nucleic Acids Res.

[31] Xu H-F, Gao X-T, Lin J-Y, Xu X-H, Hu J, Ding Y-J, Zhu S-H (2017). MicroRNA-20b suppresses the expression of ZFP-148 in viral myocarditis. Mol Cell Biochem.

[32] Ye X, Hemida MG, Qiu Y, Hanson PJ, Zhang HM, Yang D (2013). MiR-126 promotes Coxsackievirus replication by mediating cross-talk of ERK1/2 and Wnt/β-catenin signal pathways. Cell Mol Life Sci.

[33] Germano JF, Sawaged S, Saadaeijahromi H, Andres AM, Feuer R, Gottlieb RA, Sin J (2019). Coxsackievirus B infection induces the extracellular release of MiR-590-5p, a proviral microRNA. Virology.

[34] • Chen L, Hou X, Zhang M, Zheng Y, Zheng X, Yang Q, Li J, Gu N, Zhang M, Sun Y, et al. MicroRNA-223–3p modulates dendritic cell function and ameliorates experimental autoimmune myocarditis by targeting the NLRP3 inflammasome. Mol Immunol. 2020;117:73–83. 10.1016/j.molimm.2019.10.027. **This study demonstrated that miR-223–3p expression was significantly lower in experimental autoimmune myocarditis in mice. MiR-223–3p is able to inhibit inflammasome expression, promoting the polarization of dendritic cells toward a tolerogenic phenotype, with reduced function. By this mechanism, miR-223–3p effectively regulates tolerance in autoimmune myocarditis**. 10.1016/j.molimm.2019.10.027 31743855

[35] Corsten MF, Papageorgiou A, Verhesen W, Carai P, Lindow M, Obad S, Summer G, Coort SLM, Hazebroek M, van Leeuwen R (2012). MicroRNA profiling identifies microRNA-155 as an adverse mediator of cardiac injury and dysfunction during acute viral myocarditis. Circ Res.

[36] Navarro IC, Ferreira FM, Nakaya HI, Baron MA, Vilar-Pereira G, Pereira IR, Silva AMG, Real JM, De Brito T, Chevillard C, et al. MicroRNA transcriptome profiling in heart of Trypanosoma cruzi-infected mice: parasitological and cardiological outcomes. PLoS Negl Trop Dis. 2015;9: e0003828. 10.1371/journal.pntd.0003828. 10.1371/journal.pntd.0003828 PMC4473529 26086673

[37] Liu YL, Wu W, Xue Y, Gao M, Yan Y, Kong Q, Pang Y, Yang F (2013). MicroRNA-21 and -146b are involved in the pathogenesis of murine viral myocarditis by regulating TH-17 differentiation. Arch Virol.

[38] Yan L, Hu F, Yan X, Wei Y, Ma W, Wang Y, Lu S, Wang Z (2016). Inhibition of microRNA-155 ameliorates experimental autoimmune myocarditis by modulating Th17/Treg immune response. J Mol Med.

[39] Chen X, Dong S, Zhang N, Chen L, Li M-G, Yang P-C, Song J (2017). MicroRNA-98 plays a critical role in experimental myocarditis. Int J Cardiol.

[40] Sun S, Ma J, Zhang Q, Wang Q, Zhou L, Bai F, Hu H, Chang P, Yu J, Gao B (2016). Argonaute proteins in cardiac tissue contribute to the heart injury during viral myocarditis. Cardiovasc Pathol.

[41] Corsten MF, Heggermont W, Papageorgiou A-P, Deckx S, Tijsma A, Verhesen W, van Leeuwen R, Carai P, Thibaut H-J, Custers K (2015). The microRNA-221/-222 cluster balances the antiviral and inflammatory response in viral myocarditis. Eur Heart J.

[42] • Li Y, Liu X, Du A, Zhu X, Yu B. MiR‐203 accelerates apoptosis and inflammation induced by LPS via targeting NFIL3 in cardiomyocytes. J of Cellular Biochemistry. 2019;120:6605–6613. 10.1002/jcb.27955. **This experimental study explores the roles and potential mechanisms of miR-203 in myocarditis in vitro. The authors demonstrated that inhibition of miR-203 reduces cell injury induced by LPS and cell apoptosis rate, and miR-203 silencing attenuates the expression and production of inflammatory cytokines. On the contrary, overexpression of miR-203 showed the opposite trend in cell apoptosis and inflammation**. 10.1002/jcb.27955 30484891

[43] Xue YM, Chen MG, Chen DW, Wu WF, Liu YL, Lin FH (2018). The effect of microRNA-21 on myocardial fibrosis in mice with chronic viral myocarditis. Zhonghua Xin Xue Guan Bing Za Zhi.

[44] Koenecke C, Krueger A (2018). MicroRNA in T-cell development and T-cell mediated acute graft-versus-host disease. Front Immunol.

[45] Cobb BS, Nesterova TB, Thompson E, Hertweck A, O’Connor E, Godwin J, Wilson CB, Brockdorff N, Fisher AG, Smale ST (2005). T cell lineage choice and differentiation in the absence of the RNase III enzyme dicer. J Exp Med.

[46] Inácio DP, Amado T, Silva-Santos B, Gomes AQ (2018). Control of T cell effector functions by miRNAs. Cancer Lett.

[47] Muljo SA, Ansel KM, Kanellopoulou C, Livingston DM, Rao A, Rajewsky K (2005). Aberrant T cell differentiation in the absence of dicer. J Exp Med.

[48] Lu TX, Hartner J, Lim E-J, Fabry V, Mingler MK, Cole ET, Orkin SH, Aronow BJ, Rothenberg ME. MicroRNA-21 limits in vivo immune response-mediated activation of the IL-12/IFN-γ pathway, Th1 polarization, and the severity of delayed-type hypersensitivity. JI. 2011;187:3362–3373. 10.4049/jimmunol.1101235. 10.4049/jimmunol.1101235 PMC3175642 21849676

[49] Sawant DV, Wu H, Kaplan MH, Dent AL (2013). The Bcl6 target gene microRNA-21 promotes Th2 differentiation by a T cell intrinsic pathway. Mol Immunol.

[50] Murugaiyan G, da Cunha AP, Ajay AK, Joller N, Garo LP, Kumaradevan S, Yosef N, Vaidya VS, Weiner HL (2015). MicroRNA-21 promotes Th17 differentiation and mediates experimental autoimmune encephalomyelitis. J Clin Invest.

[51] O’Connell RM, Kahn D, Gibson WSJ, Round JL, Scholz RL, Chaudhuri AA, Kahn ME, Rao DS, Baltimore D (2010). MicroRNA-155 promotes autoimmune inflammation by enhancing inflammatory T cell development. Immunity.

[52] Zech A, Ayata CK, Pankratz F, Meyer A, Baudiß K, Cicko S, Yegutkin GG, Grundmann S, Idzko M (2015). MicroRNA-155 modulates P2R signaling and Th2 priming of dendritic cells during allergic airway inflammation in Mice. Allergy.

[53] Thai T-H, Calado DP, Casola S, Ansel KM, Xiao C, Xue Y, Murphy A, Frendewey D, Valenzuela D, Kutok JL (2007). Regulation of the germinal center response by microRNA-155. Science.

[54] Malmhäll C, Alawieh S, Lu Y, Sjöstrand M, Bossios A, Eldh M, Rådinger M (2014). MicroRNA-155 is essential for TH2-mediated allergen-induced eosinophilic inflammation in the lung. J Allergy Clin Immunol.

[55] •• Blanco-Domínguez, R, Sánchez-Díaz, R, de la Fuente, H, Jiménez-Borreguero, L.J, Matesanz-Marín, A, Relaño, M, Jiménez-Alejandre, R, Linillos-Pradillo, B, Tsilingiri, K, Martín-Mariscal, M.L, et al. A novel circulating microRNA for the detection of acute myocarditis. N Engl J Med. 2021;384:2014–2027. 10.1056/NEJMoa2003608. **The authors identified a novel microRNA (mmu-miR-721) as a marker of myocarditis in murine models and its human homologue (hsa-miR-Chr8:96) that could be used to distinguish patients with myocarditis from those with myocardial infarction and from healthy controls**. 10.1056/NEJMoa2003608 PMC8258773 34042389

[56] Bayraktar R, Van Roosbroeck K, Calin GA (2017). Cell-to-cell communication: microRNAs as hormones. Mol Oncol.

[57] Hagiwara S, Kantharidis P, Cooper M. MicroRNA as biomarkers and regulator of cardiovascular development and disease. CPD. 2014;20:2347–2370. 10.2174/13816128113199990495. 10.2174/13816128113199990495 23844813

[58] Siracusa J, Koulmann N, Banzet S (2018). Circulating myomiRs: a new class of biomarkers to monitor skeletal muscle in physiology and medicine: circulating myomiRs. J Cachexia Sarcopenia Muscle.

[59] Turchinovich A, Tonevitsky AG, Burwinkel B (2016). Extracellular miRNA: a collision of two paradigms. Trends Biochem Sci.

[60] Iftikhar H, Carney GE. Evidence and potential in vivo functions for biofluid miRNAs: from expression profiling to functional testing: potential roles of extracellular miRNAs as indicators of physiological change and as agents of intercellular information exchange. Bio Essays. 2016;38:367–378. 10.1002/bies.201500130. 10.1002/bies.201500130 26934338

